# Urethral Prolapse Case Report: Surgical and Social Considerations in Senegal

**DOI:** 10.1155/2022/5541416

**Published:** 2022-01-24

**Authors:** Mohamed Jalloh, Jennifer Heibig, Oumar Gaye, William Ghaul, Gabrielle Yankelevich, Medina Ndoye, Mouhamadou Moustapha Mbodji, Ayun Cassell, Lamine Niang, Serigne Magueye Gueye

**Affiliations:** ^1^Service d'Urologie, Hopital General Idrissa Pouye, 134 Rue GY, 530 Dakar, Senegal; ^2^Philadelphia College of Osteopathic Medicine (PCOM), 4170 City Ave, Philadelphia, PA 19131, USA

## Abstract

We present three cases of urethral prolapse in prepubertal females in Senegal who presented with vulvar bleeding. Careful gynecologic and urologic physical exams were performed and revealed urethral origin and prolapse. Conservative versus surgical approaches were taken in different patients, but ultimately, each patient received a urethral meatoplasty. Surgical excision of these masses yielded a full recovery in the patients. A careful review of the literature was then undertaken and showed that surgical excision or ligation of the prolapse is preferable to more conservative treatment. The case series article discusses the rare occurrence of urethral prolapse, as well as the epidemiology and prognostic and therapeutic implications of urethral prolapse in prepubertal females. *Introduction*. Urethral prolapse is a rare condition occurring mostly in young black females. It can be worrying to the parents as it often causes vulvar bleeding. *Case Presentation*. We present three cases of urethral prolapse in prepubertal females who presented with vulvar bleeding. Physical exams were performed and revealed urethral origin and prolapse. Each patient underwent a urethral meatoplasty and subsequently experienced a full recovery after respective follow-up of 2 years, 1 year, and 1 year. *Conclusion*. Urethral prolapse is a rare condition which can be managed successfully by surgery. *Plain Language Summary*. This case report on pediatric urethral prolapse showcases the different presentations and modalities of treatment, as the literature does not show that a specific treatment is always undertaken. In some countries, there are strong social considerations and they demonstrate difficulty separating sexual abuse from genitourinary pathologies, which are important to address in the treatment of these conditions.

## 1. Introduction

Urethral prolapse is rare condition in which the proximal portions of the urethra tunnel into the distal portions. The most common presenting symptom of urethral prolapse is bleeding, which is frequently misdiagnosed as sexual abuse or vaginal trauma [1]. Due to the rarity of urethral prolapse, there are no specific algorithms for treatment. We report three cases of pediatric urethral prolapse that occurred at Hospital General Idrissa Pouye (HOGIP) in Dakar, Senegal. Moreover, this series reports on associated social and medicolegal facts.

## 2. Case Report

**Figure 1:**
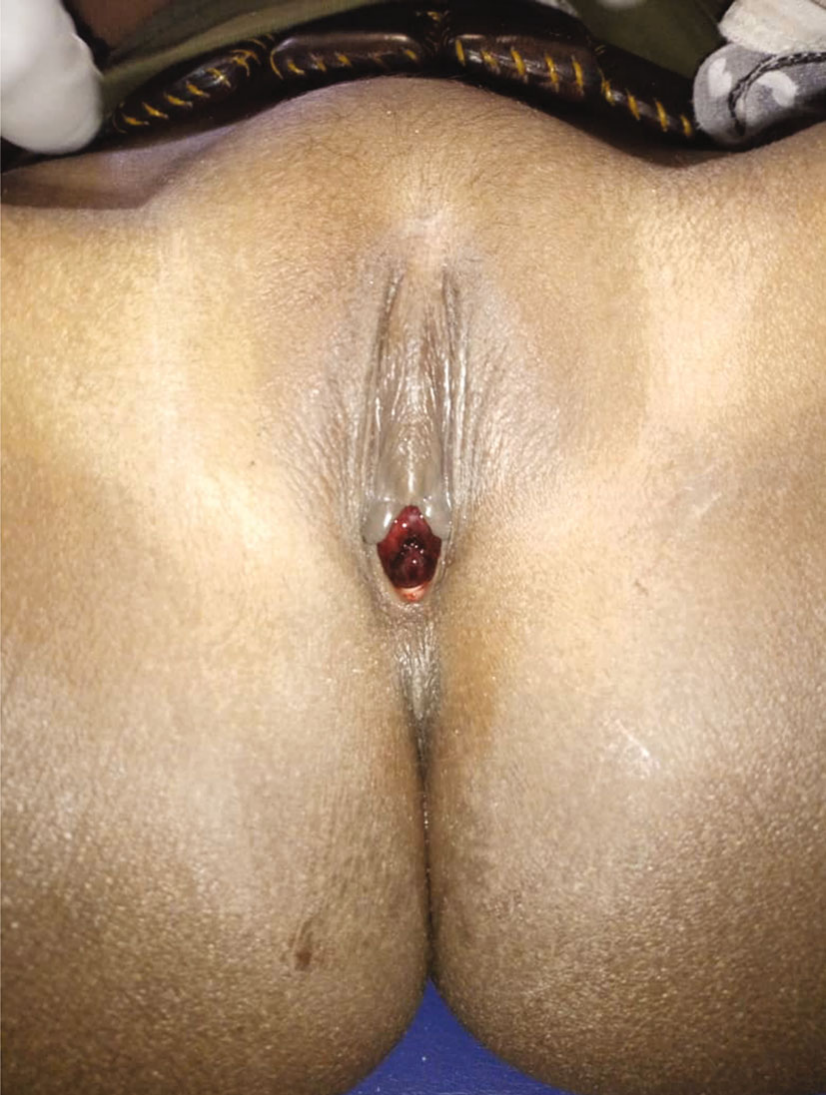
Blood at the meatus of a urethral prolapse in a 7-year-old girl.

The first case is a 7-year-old female who was referred to gynecology for a second episode of vulvar bleeding. There was a suspicion for abuse noted by the gynecologist and referring physician. On examination, the patient had bleeding only at the urethral meatus, a circular bulging mass exceeding the limit of the meatus (Figure 1) and an intact hymen and vulva. She was diagnosed with urethral prolapse. After providing parental reassurance and education, the patient was managed surgically by excision and meatoplasty. She had no acute or chronic complications at 2-year follow-up.

The second case is a 4-year-old female referred to gynecology for vulvar bleeding, she was subsequently referred to urology. The patient had an intact hymen and a circular mass exceeding the limit of the meatus without bleeding leading to a diagnosis of urethral prolapse. This patient was managed conservatively with a 2-month course of estrogen ointment, which caused a reduction in bleeding but failed to completely resolve the bleeding and reduce the meatal mass. She was managed surgically with prolapse excision and meatoplasty. The patient resolved well postoperatively and had no acute or chronic complications at a 1-year follow-up.

**Figure 2:**
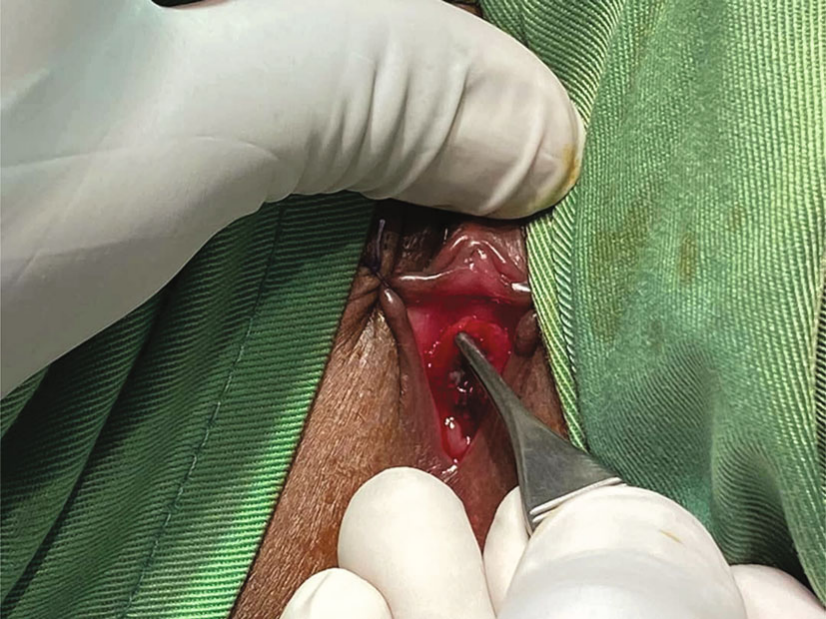
Urethral prolapse in a 7-year-old girl.

The third case is a 7-year-old female who was referred to gynecology for vulvar bleeding. She was then referred to a urologist who noted bleeding from the urethra, a mass bulging at the urethra, and a normal hymen (Figure 2). She was diagnosed with urethral prolapse and was managed with excision and meatoplasty. The postoperative course assessed 1-year postoperatively was uneventful.

## 3. Discussion

This case series brings to light several important issues surrounding urethral prolapse, a condition which affects 1 : 3000 mostly prepubertal black females or postmenopausal Caucasian females [2]. Urethral prolapse is thought to be caused by weak attachments between longitudinal and circular oblique smooth muscle layers of the urethra, which can be precipitated by pathologies that increase intra-abdominal pressure or cause ligamentous and muscular laxity [2]. Patients with asthma, upper respiratory infections, trauma, surgery, and obesity have this increase in intra-abdominal pressure [3]. Estrogen deficiency may also play a role, as estrogen normally allows for stronger pelvic floor musculature, demonstrating why this occurs in prepubertal or postmenopausal populations [3].

Important differential diagnoses, brought to light by multiple studies [1, 2, 4, 5], include ureterocele, penetrating injury, sexual abuse, foreign body, and urethral caruncle. As seen in this case series, every patient was referred to gynecology prior to urological involvement. Sexual abuse was a major concern for the physicians and parents but was absent despite the vulvar bleeding [6].

Urethral prolapse has two main treatment modalities: conservative and surgical. Conservative treatment consists mainly of local estrogen cream which targets at increasing muscle strength [2]. However, Valerie et al. [3] indicated that only 33% of patients were treated successfully with conservative management which is consistent with the findings of Jerkins et al. who found 67% of failure to medical treatment [8]. Surgical repair has two different modalities: excision or ligation, with some studies finding that excision has shorter postop hospital stays [7]. Surgical excision is performed by excising the redundant mucosa followed by anastomosis of the urethra to the vestibule [9, 10]. Surgical ligation is performed over a catheter and requires ligation of the prolapsed tissue. Surgical management has dramatically reduced recurrence rates compared to conservative management [11]. Supporting the current literature, all 3 patients in this series underwent surgical management with prior estrogen treatment in 1 case.

As seen in the first case, there was great parental concern regarding the possibility of sexual abuse. Taking that fact into consideration adds to the current body of knowledge in the literature. Therefore, patient education and reassurance are important.

## 4. Conclusion

Although rare, urethral prolapse is noticeable in the black pediatric population and has excellent outcomes when treated surgically. An interdisciplinary approach and a thorough differential are necessary in order to properly identify and treat urethral prolapse. Surgical management remains the gold standard.

## Data Availability

Data are available in the medical records of patients at Hospital General Idrissa Pouye.
